# Molecular Subtyping in Muscle-Invasive Bladder Cancer on Predicting Survival and Response of Treatment

**DOI:** 10.3390/biomedicines11010069

**Published:** 2022-12-27

**Authors:** Tanan Bejrananda, Jirakrit Saetang, Surasak Sangkhathat

**Affiliations:** 1 Department of Surgery, Faculty of Medicine, Prince of Songkla University, Songkhla 90110, Thailand; 2 International Center of Excellence in Seafood Science and Innovation, Faculty of Agro-Industry, Prince of Songkla University, Songkhla 90112, Thailand; 3 Department of Biomedical Sciences and Biomedical Engineering, Faculty of Medicine, Prince of Songkla University, Songkhla 90110, Thailand; 4 Translational Medicine Research Center, Faculty of Medicine, Prince of Songkla University, Songkhla 90110, Thailand

**Keywords:** molecular subtypes, muscle-invasive bladder cancer, the cancer genome atlas (TCGA), transcriptomic analysis

## Abstract

Molecular classifications for urothelial bladder cancer appear to be promising in disease prognostication and prediction. This study investigated the novel molecular subtypes of muscle invasive bladder cancer (MIBC). Tumor samples and normal tissues of MIBC patients were submitted for transcriptome sequencing. Expression profiles were clustered using K-means clustering and principal component analysis. The molecular subtypes were also applied to The Cancer Genome Atlas (TCGA) dataset and analyzed for clinical outcome correlation. Three molecular subtypes of MIBC were discovered, clusters A, B, and C. The most differentially upregulated genes in cluster A were BDKRB1, EDNRA, AVPR1A, PDGFRB, and TNC, while the most upregulated genes in cluster C were collagen-related genes, PDGFRB, and PRKG1. For cluster B, COL6A3, COL1A2, COL6A2, tenascin C, and fibroblast growth factor 2 were statistically suppressed. When the centroids of clustering on PCA were applied to TCGA data, the clustering significantly predicted survival outcomes. Cluster B had the best overall survival (OS), and cluster C was associated with poor OS but exhibited the best response to perioperative chemotherapy. Among all groups, cluster B had a better pathologic response to neoadjuvant chemotherapy (40%). Based on the results of the present study, the novel clusters of subtype MIBC appear potentially suitable for integration into clinical practice.

## 1. Introduction

Bladder cancer (BC) is ranked as the ninth most frequently diagnosed cancer worldwide. It is mainly represented by bladder urothelial carcinoma, which accounts for 90% of BC [1]. Current standard treatments for BC mainly include surgical resection, followed by chemotherapy; however, due to the high incidence of distant metastasis and recurrence after treatment, the five-year overall survival rate remains at 15–20% [2]. High morbidity, mortality, and healthcare costs associated with muscle-invasive bladder cancer (MIBC) draw a need for personalized patient care [3]. Recent advances in molecular profiling have been proposed in individualizing treatment for MIBC patients [4]. While the divergent but interrelated two-molecular-pathway model for the development of low-grade and high-grade BC is well-established, molecular profiling has revealed heterogeneity in the genetic landscape within MIBC, which also reflects its diversity in clinical outcomes [5,6,7].

Transcriptome profiling studies from different institutions, including The Cancer Genome Atlas (TCGA) research network, have identified distinct molecular subtypes within MIBC [8,9,10,11]. Different studies have proposed varying MIBC subtyping schemes such as the TCGA clusters, Lund Taxonomy, mRNA expression-based molecular subtypes (TCGA-2017), and the consensus subtypes produced by the Bladder Cancer Molecular Taxonomy Group (Consensus) [9,10,11,12,13,14,15,16]. At the highest level of hierarchy, MIBC can be divided into two major subtypes—“basal” (BL) and “luminal” (LU). The BL subtype shares an expression profile with the basal cells of the urothelium and is usually associated with a poor prognosis. The LU subtype shares molecular profiles with differentiated urothelial cells and generally predicts better prognosis [7,9,10,13,14]. In the original Lund Taxonomy classification from 2012, the best cancer-specific survival (CSS) was associated with the “UroA” subtype (now “Urothelial-like”), which represented low-grade non-MIBC with a signature similar to the LU subtype [17]. Intra-tumor and intra-patient co-existence of basal and luminal tumor regions has been reported in MIBC patients, and there were also non-MIBC tumors that exhibited expression profiles similar to MIBC subtypes [13,18]. In addition, intratumoral heterogeneity which may be enhanced following neoadjuvant and adjuvant treatment can be responsible for the fluidity of molecular profiling patterns [18,19].

The primary focus of this study was to develop and validate a subtyping that could be readily applied to our patient cohort for predicting clinical outcome. We initially subtyped our institutional MIBC cohort using an unsupervised clustering of transcriptomic expression profiling. The clusters were validated with TCGA dataset of MIBC [4,11,12,14,20,21,22]. 

## 2. Materials and Methods

### 2.1. Sample Selection and RNA Isolation 

Frozen tissue samples from 30 consecutive MIBC patients who underwent radical cystectomy at Songklanagarind Hospital, Thailand, from 2015 to 2020, and 7 normal bladder mucosa tissue samples from patients with hematuria but without bladder cancer were studied. Representative sections from all specimens were re-evaluated for their histopathology by a pathologist (K.K.). Regarding specimen collection and storage, tissues were collected at the time of surgical resection, snapped into the size of around 0.5 cm, stored in a cryotube with stabilization solution (RNAlater, Thermo Fisher Scientific, Inc., Waltham, MA, USA), and kept frozen in liquid nitrogen until RNA extraction. Seven samples of non-tumorous urinary bladder wall obtained during a cystectomy were used as controls. Informed consent was obtained from all patients, and the study was approved by the Ethical Committee of Songklanagarind Hospital, Prince of Songkla University, in accordance with the Declaration of Helsinki (REC 61-222-10-1). 

RNA was isolated from all samples with the DNA/RNA AllPrep kit (QIAGEN). Extracted RNA was assessed for quantity using Nanodrop 1000 (Nanodrop) or Qubit (Thermo Fisher Scientific, Waltham, MA, USA). Digital quality control (QC) analysis for the integrity of total RNA was performed using the 2100 Bioanalyzer (Agilent Technologies, Quebec City, QC, Canada), following the manufacturer’s instructions. 

### 2.2. Gene Expression Study

From each RNA sample, 3 ug of total RNA was used for strand-specific library preparation. Illumina Stranded mRNA preparation kit (Illumina) was used to generate the sequencing libraries according to the manufacturer’s protocol. cDNA was prepared with random hexamer primer. The Illumina NovaSeq 6000 platform was used for transcriptome sequencing following the manufacturer’s instructions.

Paired-end raw data in FASTQ format from the sequencing machine were checked for read quality, size, and GC content using the FASTQC program. Reads were aligned to the reference genome using STAR version 2.7.8. The total mapping rate and mapped read number were analyzed using HTSeq version 0.13.5. The total number of mapped reads and fragments per kilobase of exon model per million mapped reads (FPKM) were calculated for each annotated gene. DESeq2 package (R program) was used to capture the DEG. The differentially expressed genes (DEGs) for 30 BC samples and 7 non-tumorous bladder tissue samples were analyzed with the DESeq2 package, and |log2FC| > 2 and *p* < 0.05 were set as the cutoff for DEGs.

### 2.3. Clustering and Differential Expression Analysis

The transcriptomic data from MIBC samples were then clustered by K-means clustering and the principal component analysis method. Differentially expressed genes among clusters were performed and shown as a Venn diagram. Differentially expressed genes among clusters were analyzed for signaling pathway enrichment in each cluster using KEGG (Kyoto Encyclopedia of Genes and Genomes) term enrichment analysis, using a cutoff *p*-value of 0.05. Genes that showed different expression levels in all clusters were evaluated for their diagnostic performance by using Receiver Operating Curve (ROC) analysis to evaluate the performance of expression levels in predicting the cluster of MIBC. 

### 2.4. Validation with TCGA Dataset

The selection of TCGA data used the keyword “muscle invasive bladder cancer”. Clinicopathological data on the cohort and mRNA data were downloaded from the open access portal, cBioPortal (https://www.cbioportal.org, accessed on 24 March 2022). Patients with an unknown tumor stage (Tx) or T < 2 were excluded from analysis, yielding a final study population of 231 TCGA patients. Statistical analyses were performed using R program version 4.1.10. Association between subtype and clinical outcome was analyzed by univariate (single parameter logistic regression) analysis. ROC curves were used to compute sensitivity and specificity. Mean, median, and 95% confidence interval (CI) of sensitivity and specificity were calculated. 

Association between subtypes and time to metastasis, recurrence-free survival, cancer-specific survival, or OS was determined while adjusting for demographic and clinical covariates using the Cox proportional hazard model with a stepwise selection procedure. Kaplan–Meier plots with log-rank statistics determined if subtypes classified MIBC patients into risk categories based on survival outcome. Chi-square tests were used to compare clinicopathologic data of patients for different amplicon definitions. Comparisons included age, gender, tumor stage, lymph node status, and molecular subtype. Kaplan–Meier plots with log-rank statistics categorized MIBC patients into outcome risk categories. Molecular subtypes and age were compared. The Bonferroni adjustment was employed to correct for multiple testing. The significance of univariable Kaplan–Meier regressions was assessed using the log-rank and Wilcoxon tests. Multivariable analyses were conducted using Cox proportional hazard regression. For results from the univariable analysis, a *p*-value cutoff of <0.2 was chosen to include relevant clinical or pathological parameters that would have been missed with a more restrictive *p*-value of <0.05. Contingency analyses of nominal variables were performed with Pearson’s chi-squared test. Variables for the multivariable analysis included significant (*p* < 0.2) clinicopathological characteristics on univariable analysis (pT-Stage, pN-Stage, age, gender) and genes. Statistical analyses of numeric continuous variables were performed with non-parametric tests (Wilcoxon rank-sum test, Kruskal–Wallis test).

## 3. Results

### 3.1. Patient Information and Clinical Characteristics

All 30 tissue samples were from patients recruited at Songklanagarind Hospital, Songkhla, Thailand. These included tumor tissue from 26 males and 4 females with ages between 52 and 92 years. Clinical data are provided in Table 1. The data of 231 MIBC cohorts retrieved from The Cancer Genome Atlas (TCGA) are also shown in Table 1 and include the clinical information from 169 males and 42 females between the ages of 46 and 90 years old. It should be noted that there is a quite difference in the proportion of T stages and N stages between data from tissue samples and the TCGA cohort. Moreover, no metastasis was found in our MIBC patients.

### 3.2. Transcriptome Profiling and Classification of Thai MIBC

**Figure 1:**
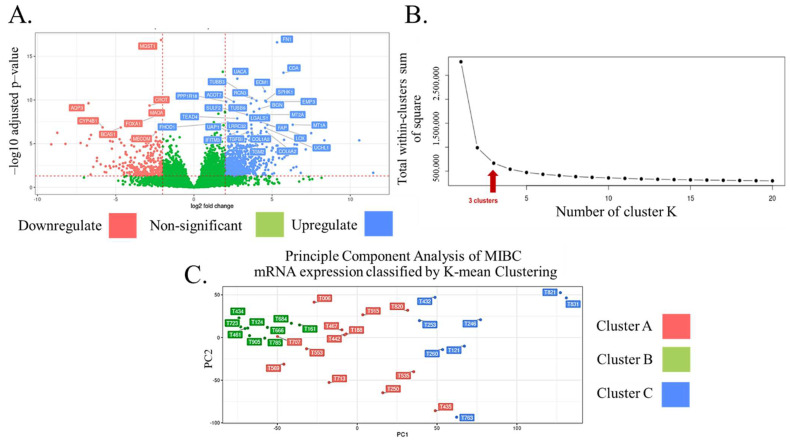
(**A**) Volcano plot analysis demonstrated that more than 100 genes were found to be upregulated and downregulated in MIBC. (**B**) Elbow plot analysis was applied for Thai MIBC transcriptome data. The results show that the three clusters were found to be optimal. (**C**) The transcriptomic data of all MIBC tissue samples were then subjected to the classification of the MIBC groups by using principal component analysis by K-means clustering. The genes were ranked by adjusted *p*-value.

The transcriptome sequencing of all tissue samples was performed based on the strand-specific library preparation to identify the expression levels of all genes. Differential expression analysis and a volcano plot demonstrated that 544 genes were found to be upregulated and downregulated in MIBC (Figure 1A and Table S1). According to their ontology, the 30 most significantly changed genes included phosphatidylinositol 3-kinase (PI3K)/protein kinase B (AKT) signaling molecules (fibronectin 1 (FN1), collagen type VI alpha 2 chain (COL6A2), and collagen type I alpha 2 chain (COL1A2)), mitogen-activated protein kinase (MAPK) pathway-related molecules (transforming growth factor-beta 1 (TGFB1) and MDS1 and EVI1 complex locus (MECOM)), mitochondrial biogenesis regulators (metallothionein 1A (MT1A) and MT2A), exosomal proteins (tubulin beta 6 class V (TUBB6), TUBB3, galectin 1 (LGALS1), and interferon-induced transmembrane protein 3 (IFITM3)), biomolecule metabolism (cytidine deaminase (CDA), sphingosine kinase 1 (SPHK1), monoamine oxidase A (MAOA), and microsomal glutathione S-transferase 1 (MGST1)), and others. To identify the optimal number of clusters based on transcriptomic classification, Elbow plot analysis was applied for Thai MIBC transcriptome data. The results show that the three clusters were found to be optimal, as demonstrated in Figure 1B. The transcriptomic data of all MIBC tissue samples were then subjected to the classification of the MIBC groups by using principal component analysis by K-means clustering.

**Figure 2:**
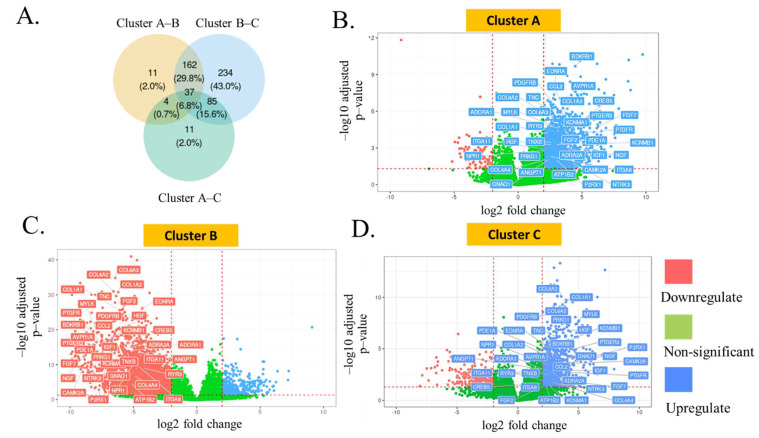
(**A**) The data from differential gene expression (DEG) analysis also revealed the number of genes that expressed differently between two clusters, as displayed in the Venn diagram. The expression levels of each gene are demonstrated as volcano plots for clusters A (**B**), B (**C**), and C (**D**).

In addition to the enrichment study, the data from differential gene expression (DEG) analysis also revealed the number of genes that were expressed differently between two clusters, as displayed in the Venn diagram (Figure 2A). These included the genes related to the calcium signaling pathway (bradykinin receptor B1 (BDKRB1), endothelin receptor type A (EDNRA), arginine vasopressin receptor 1A (AVPR1A), prostaglandin E receptor 3 (PTGER3), prostaglandin F receptor (PTGFR), neurotrophic receptor tyrosine kinase 3 (NTRK3), purinergic receptor P2X 1 (P2RX1, etc.), PI3K-Akt signaling pathway (COL6A2, COL1A2, integrin subunit alpha 8 (ITGA8), CAMP responsive element binding protein 5 (CREB5), COL6A3), MAPK signaling pathway (fibroblast growth factor 7 (FGF7), nerve growth factor (NGF), hepatocyte growth factor (HGF), angiopoietin 1 (ANGPT1)), or cyclic GMP-dependent protein kinase (cGMP-PKG) signaling pathway (potassium calcium-activated channel subfamily M regulatory beta subunit 1 (KCNMB1), KCNMA1, adrenoceptor alpha 2A (ADRA2A), ATPase Na^+^/K^+^ transporting subunit beta 2 (ATP1B2), adenosine A1 receptor (ADORA1), protein kinase cGMP-dependent 1 (PRKG1)). The expression levels of each gene were demonstrated as volcano plots for each cluster (Figure 2B–D). Interestingly, all 37 genes were significantly upregulated in clusters A and C, but downregulated in cluster B. The most significantly expressed genes in cluster A included BDKRB1, EDNRA, AVPR1A, platelet-derived growth factor receptor beta (PDGFRB), and tenascin C (TNC), while COL6A3, COL1A1, COL6A2, PDGFRB, and PRKG1 were found to be the top five genes highly expressed in cluster C. For cluster B, the collagen-related genes (COL6A3, COL1A2, COL6A2), TNC, and fibroblast growth factor 2 (FGF2) were the statistically suppressed transcripts.

### 3.3. ROC Analysis of 37 Differentially Expressed Genes Found in MIBC Tissues

To evaluate the specificity and sensitivity of the genes expressed differently for each MIBC cluster, ROC curve analysis was performed for all 37 genes and all clusters. 

Interestingly, the corresponding areas under the ROC curve (AUCs) with values more than 0.8, 0.9, and 0.95 were found in 33, 25, and 14 genes from 37 genes for cluster B (Table 2 and Figure S1). The AUC values above 0.9 were observed only in five genes for cluster C; none could be found for cluster A. The highest AUC value of cluster B was 0.988 for PDGFRB and COL6A2 genes; meanwhile, the lowest AUC was 0.72 for the ITGA8 gene (Table 2 and Figure S1). KCNMB1 is the gene that presented the highest AUC with the value of 0.936 in cluster C while ITGA11 showed the lowest AUC with 0.67. Cluster A displayed the lowest value of mean AUC; the range of AUC values of differentially expressed genes in this cluster was between 0.52 and 0.869, with the ryanodine receptor 3 (RYR3) gene showing the lowest and COL1A1 the highest AUC.

### 3.4. Clinical Characteristics and Molecular Subtypes of MIBC Associated with Treatment Outcome and the Response of Perioperative Chemotherapy

To identify factors associated with MIBC, logistic regression analysis was performed, including prognostic scores, patient characteristics, and tumor characteristics (Table 1). Univariate analysis identified tumor stage and nodal status as significant predictors of overall survival. The multivariate logistic regression model identified the tumor stage (hazard ratio (HR), 25.64; 95% confidence interval (CI), 2.31–284.04; *p* = 0.006) as an independent predictor of overall survival. Cluster C exhibited a higher hazard ratio without being statistically significant (HR, 2.63; 95% CI, 0.44–15.79; *p* = 0.291) (Table 3).

**Figure 3:**
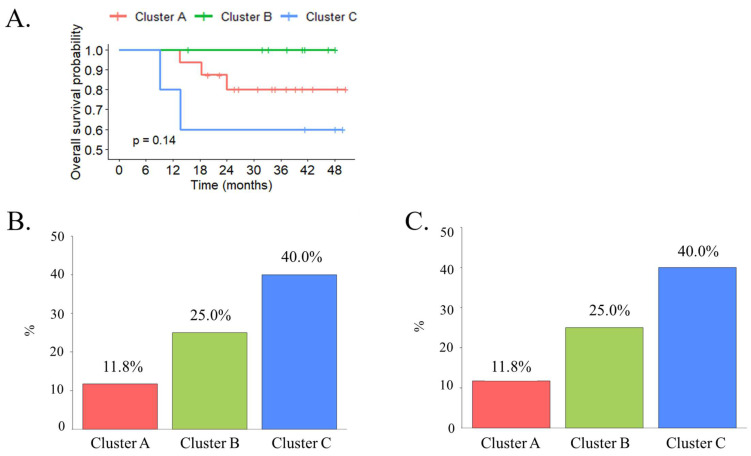
(**A**) The Kaplan–Meier analysis demonstrated that cluster B displayed the highest probability of survival within 48 months of follow-up while cluster C showed the lowest value. (**B**) RNA-based molecular subtypes are associated with pathologic response to neoadjuvant chemotherapy. (**C**) RNA-based molecular subtypes are associated with pathologic response to adjuvant chemotherapy.

Univariate analysis showed no significant differences in survival in our patients between the molecular subtypes (*p* = 0.108). Pairwise comparisons using log-rank tests also showed that the overall survival did not differ significantly between each molecular subtype, with patients with the cluster B subtype experiencing the longest survival, followed by those with the cluster A subtype. The poorest survival was observed among patients with the cluster C subtype (Figure 3A).

We applied molecular subtype classification to tumors from 30 patients treated with preoperative chemotherapy and analyzed the response to neoadjuvant chemotherapy. Among these patients, cluster C had a better pathologic response to neoadjuvant chemotherapy (40%) (Figure 3B). Moreover, MIBC in cluster C also exhibited better outcomes after adjuvant chemotherapy for progression or metastatic-free survival (Figure 3C).

### 3.5. The Transcriptomic Classification Using PCA Analysis of Tissue Samples with the TCGA Data Provided a Significant Prognostic Value of MIBC Overall Survival

**Figure 4:**
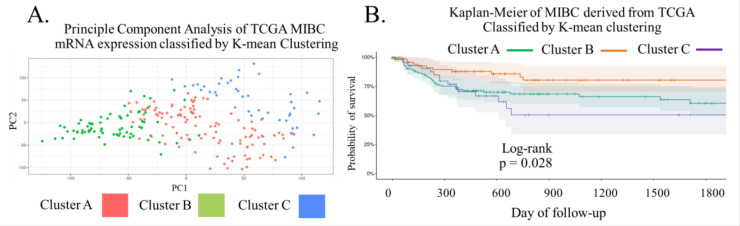
(**A**) The centroid was derived from MIBC tissue samples to separate PCA coordinates of the TCGA cohort into three clusters according to MIBC tissues. (**B**) The Kaplan–Meier analysis demonstrated that cluster B displayed the highest probability of survival within 1800 days of follow-up, while cluster C showed the lowest value.

To increase the number of samples used in this study, we included 231 transcriptomic data from TCGA database for classification by using PCA analysis and unsupervised K-means clustering. We decided to apply the centroid derived from MIBC tissue samples to separate PCA coordinates of the TCGA cohort into three clusters according to MIBC tissues (Figure 4A). We also determined the relationship between each cluster and survival data. The Kaplan–Meier analysis demonstrated that cluster B displayed the highest probability of survival within 1800 days of follow-up while cluster C showed the lowest value (*p* = 0.028; Figure 4B). Clinical characteristics of MIBC from TCGA in each cluster are shown in Table S2.

### 3.6. Certain Signaling Pathways Were Associated with Each Type of MIBC Cluster

To identify the signaling pathways enriched in each cluster, the transcriptomic data were used with KEGG (Kyoto Encyclopedia of Genes and Genomes) term enrichment analysis. The metabolic pathways or signal transduction pathways associated with differentially expressed genes, comparing the whole genome background with the KEGG terms and *p*-adj < 0.05, are justified as significant enrichment. The top 10 significantly enriched terms in the KEGG enrichment analysis are displayed for each cluster (Table 4). The calcium signaling pathway was found to be a generally significant process in all clusters. However, the chemokine signaling pathway was observed significantly only in clusters A and B. Interestingly, the immune signal transduction pathways, including Janus kinase-signal transducer and activator of transcription (JAK-STAT), B cell receptor signaling, and T cell receptor signaling pathways, were marked as the key mechanisms in cluster A of MIBC specifically, while advanced glycation end product-receptor for AGE (AGE-RAGE) and Ras-associated protein-1 (Rap1) signaling pathways were found as significantly enriched molecular processes in cluster B. For cluster C, cGMP-PKG, oxytocin, MAPK, and Relaxin signaling pathways were observed to be unique in this cluster.

## 4. Discussion

MIBC is deadly but curable if treated properly. Therefore, precise classification of molecular subtypes responsible for treatment outcomes is important. Nowadays, many studies have reported the MIBC subtype according to next-generation sequencing (NGS) profiling, such as the TCGA clusters, Lund Taxonomy, mRNA expression-based molecular subtypes (TCGA-2017), and the consensus subtypes produced by the Bladder Cancer Molecular Taxonomy Group [9,10,11,12,13,14,15,16]. However, molecular classification in MIBC still has heterogeneity because of diversity in the genetic background, clinical treatment, and clinical outcome [5,6,7].

In addition, most available clustering schemes are based on cDNA microarray data, and the current high-throughput molecular techniques have migrated to the next-generation sequencing platform.

To our knowledge, our study is the first report of a single institutional MIBC subtyping cohort using unsupervised clustering based on transcriptomic data. The primary finding of this study is that the locations of MIBC clusters on the principal components identified from transcriptome data can be predicted from an understanding of the average coalescent differential genes for tissue samples. This analysis transformed the high-dimensional data into an orthogonal basis which represents the variants of mRNA expression profile in each sample. Unsupervised clustering revealed the three clusters of MIBC with the 37 genes expressed differently in all clusters. Interestingly, all these genes are related to the signaling pathway associated with cancers. For example, the calcium signaling pathway was found to be increased in colon cancer [23], breast cancer [24], and liver cancer [25]. PI3K-Akt activation was also found in breast cancer [26], gastric cancer [27], and thyroid carcinoma [28]. The ubiquitous signal transduction MAPK pathway is also associated with cancer cell proliferation and survival and an inflammatory environment [29]. Although all these signal transductions are in cancers, the dominant pathway in the cancer cell depends on the genetic background, the mutation status, or type of cancer [30], determining the aggressive behavior, the progression rate, and drug response of cancer. 

Surprisingly, most of the genes are not related to the markers used for sub-typing in the previous reports [4,9,10,11,12,13,14,15,16]. When compared to the original report that used data from the Dana-Faber Cancer Institute as a training set, only two markers in our study (MT2A, uniquely expressed in cluster A, and HMGCS2, uniquely expressed in the cluster B) overlapped with the BASE47 panels of that work [9]. The difference in molecular patterns that defined sub-types might be explained by the difference in the expression study technique, as our study used transcriptomic profiling by RNA sequencing rather than cDNA microarray. Among the 37 genes that were significantly less differentially expressed in our cluster B, 13 had differential expression levels in that study, and all 13 were overexpressed in their Basal subtype, which means that our cluster B corresponded to the Luminal subtype. Consistent with other studies in which the Basal subtype had poorer prognosis, our cluster B had higher overall survival probability, which was also validated by TCGA data. Apart from transcriptomic profiling, other studies evaluated the association of other biological factors such as lncRNA, miRNA, protein expression, or DNA methylation [9,10,11,12,13,14,15,16], while we focused only on transcriptomic profiles and determined the sensitivity and specificity of the genes instead. ROC curve analysis revealed that most of the genes showed a high correlation of sensitivity and specificity only for cluster B, which may be used as expression markers for good prognosis in Thai MIBC patients. Our transcriptomic clustering provided three clusters of MIBC tissue which expressed the specific pattern of mRNA profiling. In addition to our MIBC transcriptomic study from patients, we used the information from TCGA dataset for validation. However, PCA analysis with the comparison between two cohorts demonstrated the obviously different PC coordinates between data from our MIBC tissue samples and TCGA dataset. The variation in the genetic background of the different populations studied may be the factor that caused the difference in the PCA data plot [31]. By using the initial cluster centroids of the MIBC tissue data, we applied the distance of tissue PCA coordinated with mRNA expression profile to TCGA data for transcriptomic clustering of the TCGA cohort. This confirmed the influence of distinct expression scenarios on other populations. The three clusters obtained from this method were related to the significant difference in the overall survival of MIBC patients, meaning that the classification based on transcriptomic data of MIBC tissue may be an alternative way to predict the survival outcome. 

Historically, molecular classification of bladder tumors has identified two divergent pathways with distinct genetic hallmarks characterizing low-grade and high-grade tumors. Molecular subtyping classifications have provided insight into the biology of bladder tumors. It was reported that MIBC subtypes may predict patient response to chemotherapy and could be used to develop targeted therapies. Since gene expression patterns change over the course of treatment, subtype drifting or switching due to tumor evolution and reaction to therapy should be concerned. Therefore, there is a need for systematic biological studies to better stratify BC based on genetic drivers of treatment response rather than subtyping based on expression profiling alone [10,32]. 

The present study demonstrates that subtyping by an unsupervised differential gene expression based on RNA-Seq is possibly useful in predicting survival outcomes. Furthermore, regardless of subtyping classification, only clinical parameters such as N-stage and LVI were independent predictors of prognosis in multivariate analyses. In any cohort, subtypes by any classification may provide suboptimal efficacy to predict the clinical outcome if not used together with clinical parameters. The limitation of our study was the relatively low number of BC samples used in the transcriptomic analysis. In addition, most clustering to date uses multi-omics platforms which combine genome variants with expression data [33]. In the present study, although the clustering could be validated with the TCGA dataset, the method should be further evaluated in an adequate number of Thai MIBC to narrow down the representative markers for clinical application. Recent translational research focused on using non-invasive biomolecular markers such as urinary biomarkers or blood profiles in the diagnosis and classification of BC [34,35,36]. In combination with transcriptomic profiling data, more precise stratification of patient care in BC can be expected in the near future.

## 5. Conclusions

Molecular subtyping classifications have provided insight into the biology of bladder tumors, especially regarding the relationship between tumor heterogeneity and prognosis. Results from multiple cohorts and our classification systems revealed that subtypes are strongly associated with histopathologic grade and are consistent with clinical parameters to predict BC patient outcomes. Cluster B in our study had a significantly higher survival probability with the current standard treatment. However, a disparity in subtyping consensus between studies exists and needs verification by modern high-throughput genotyping techniques. Further investigation is needed to find the clinical applicability of molecular subtypes before their incorporation into the personalized care of MIBC patients.

## Figures and Tables

**Table 1:** Clinical data summary of studied MIBC datasets.

	Thai Patient Dataset	Percentage	TCGA Dataset	Percentage
Samples	30		231	
Average age (range)	67.5 (52–92)		69 (46–90)	
Gender Male Female	26 4	86.2 13.8	169 62	73.16 26.64
ECOG 0 1	21 9	70 30	158 73	68.5 31.5
T stage T 2 T 3 T 4	24 6 0	80 20 0	75 123 33	32.47 53.25 14.28
N stage N 0 N 1 N 2 N 3 N x	22 7 1 0 0	73.3 23.3 3.3 0 0	143 28 42 4 14	58.01 10.68 18.2 1.94 6.06
M stage M 0 M 1 Not available	30 0 0	100 0 0	116 5 110	50.22 2.16 47.62

**Table 2:** The area under the curve (AUC) from specificity and sensitivity of the gene expression level to define each MIBC cluster.

Genes	Cluster A	Cluster B	Cluster C
CCL2 (C-C Motif Chemokine Ligand 2)	0.7680	0.941	0.859
FGF2 (Fibroblast growth factor 2)	0.6940	0.947	0.886
RYR3 (Ryanodine receptor 3)	**0.5200**	0.721	0.735
MYLK (Myosin light chain kinase)	0.8040	0.955	0.914
EDNRA (Endothelin receptor type A)	0.8110	0.951	0.833
FGF7 (Fibroblast growth factor 7)	0.7890	0.976	0.927
BDKRB1 (Bradykinin receptor B1)	0.7030	0.872	0.727
PDGFRB (Platelet-derived growth factor receptor beta)	0.8380	**0.988**	0.838
HGF (Hepatocyte Growth Factor)	0.7310	0.945	0.867
AVPR1A (Arginine vasopressin receptor 1A)	0.6200	0.774	0.708
NGF (Nerve growth factor)	0.6650	0.893	0.783
PTGFR (Prostaglandin F receptor)	0.7230	0.947	0.912
PDE1A (Phosphodiesterase 1A)	0.6170	0.916	0.86
PTGER3 (Prostaglandin E receptor 3)	0.7790	0.951	0.821
CAMK2A (Calcium/calmodulin-dependent protein kinase 2 Alpha)	0.8200	0.971	0.862
NTRK3 (Neurotrophic receptor tyrosine kinase 3)	0.5380	0.828	0.831
P2RX1 (Purinergic receptor P2X 1)	0.7710	0.96	0.907
TNC (Tenascin C)	0.8530	0.947	0.746
COL4A4 (Collagen type IV alpha 4 chain)	0.5890	0.852	0.785
COL6A2 (Collagen type VI alpha 2 Chain)	0.8640	**0.988**	0.886
ITGA8 (Integrin subunit alpha 8)	0.6030	**0.72**	0.799
COL6A3 (Collagen type VI alpha 3 chain)	0.8430	0.979	0.849
CREB5 (CAMP-responsive element binding protein 5)	0.8200	0.946	0.79
TNXB (Tenascin XB)	0.6460	0.875	0.815
ANGPT1 (Angiopoietin 1)	0.6820	0.826	0.686
IGF1 (Insulin-like growth factor 1)	0.689	0.919	0.879
COL1A1 (Collagen type I alpha 1 chain)	**0.869**	0.95	0.736
COL1A2 (Collagen type I alpha 2 chain)	0.857	0.967	0.787
ITGA11 (Integrin subunit alpha 11)	0.824	0.915	**0.67**
NPR1 (Natriuretic peptide receptor 1)	0.628	0.885	0.808
KCNMB1 (Potassium calcium-activated channel subfamily M regulatory beta subunit 1)	0.749	0.957	**0.936**
ADORA1 (Adenosine A1 receptor)	0.761	0.89	0.762
PRKG1 (Protein kinase cGMP-dependent 1)	0.777	0.964	0.889
ATP1B2 (ATPase Na^+^/K^+^ transporting subunit beta 2)	0.542	0.743	0.751
ADRA2A (Adrenoceptor alpha 2A)	0.582	0.915	0.845
KCNMA1 (Potassium calcium-activated channel subfamily M alpha 1)	0.799	0.954	0.88
GNAO1 (G Protein subunit alpha O1)	0.739	0.939	0.85

**Table 3:** Univariate and multivariate survival association studies by Cox hazard analysis of our MIBC patients.

Variables	Univariate Analysis			Multivariate Analysis		
	HR	95% Cl	*p* Value	HR	95% Cl	*p* Value
**T stage**						
**2**	Ref					
**3–4**	6.62	1.09–40.1	0.041	25.64	2.31–284.04	0.006
**N stage**						
**0**	Ref		0.036			
**1**	4.05	0.45–5.46				
**2**	6.46	0.35–6.86				
**Age (years)**						
**≤65**	Ref					
**>65**	2.42	0.27–21.67	0.392			
**Lymph node metastasis**						
**negative**	Ref					
**positive**	2.39	1.29–4.41	0.01			
**LVI**						
**negative**	Ref					
**positive**	1.08	0.18–6.5	0.929			
**Ureteric margin**						
**negative**	Ref					
**positive**	5.32	0.59–48.17	0.212			
**Cluster**						
**Cluster A**	Ref					
**Cluster B**	0	1.6–4.94	0.108			
**Cluster C**	2.63	0.44–15.79	0.291			

**Table 4:** The top 10 significantly enriched terms in the KEGG enrichment analysis in each cluster.

No.	Term	Overlap	*p*-Value	Adjusted *p*-Value
Cluster A				
1	Chemokine signaling pathway	44/192	2.24 × 10^−10^	9.16 × 10^−9^
2	Calcium signaling pathway	49/240	1.50 × 10^−9^	5.38 × 10^−8^
3	JAK-STAT signaling pathway	32/162	2.14 × 10^−6^	4.08 × 10^−5^
4	PI3K-Akt signaling pathway	55/354	2.45 × 10^−6^	4.38 × 10^−5^
5	B cell receptor signaling pathway	18/81	7.13 × 10^−5^	7.03 × 10^−4^
6	Ras signaling pathway	35/232	2.80 × 10^−4^	2.28 × 10^−3^
7	T cell receptor signaling pathway	19/104	6.66 × 10^−4^	4.76 × 10^−3^
8	cGMP-PKG signaling pathway	26/167	1.00 × 10^−3^	6.85 × 10^−3^
9	Rap1 signaling pathway	29/210	3.48 × 10^−3^	2.16 × 10^−2^
10	NF-kappa B signaling pathway	17/04	4.24 × 10^−3^	2.58 × 10^−2^
Cluster B				
1	Calcium signaling pathway	94/240	2.89 × 10^−16^	1.73 × 10^−14^
2	PI3K-Akt signaling pathway	112/354	1.09 × 10^−11^	4.11 × 10^−10^
3	Chemokine signaling pathway	71/192	2.95 × 10^−8^	9.77 × 10^−10^
4	cGMP-PKG signaling pathway	57/167	6.91 × 10^−8^	9.45 × 10^−6^
5	AGE-RAGE signaling pathway in diabetic complications	39/100	1.58 × 10^−7^	1.98 × 10^−6^
6	Rap1 signaling pathway	62/210	5.47 × 10^−6^	4.71 × 10^−5^
7	cAMP signaling pathway	63/126	7.13 × 10^−6^	5.80 × 10^−5^
8	Ras signaling pathway	65/232	2.14 × 10^−5^	1.61 × 10^−4^
9	Apelin signaling pathway	41/137	1.43 × 10^−4^	9.18 × 10^−4^
10	Phospholipase D signaling pathway	43/148	2.08 × 10^−4^	1.31 × 10^−3^
Cluster C				
1	cGMP-PKG signaling pathway	32/167	2.30 × 10^−9^	1.25 × 10^−7^
2	Calcium signaling pathway	38/240	1.96 × 10^−8^	5.92 × 10^−7^
3	PI3K-Akt signaling pathway	46/354	3.13 × 10^−4^	8.52 × 10^−6^
4	Oxytocin signaling pathway	21/154	2.53 × 10^−4^	4.06 × 10^−3^
5	MAPK signaling pathway	33/294	2.54 × 10^−4^	4.06 × 10^−3^
6	cAMP signaling pathway	26/216	3.81 × 10^−4^	5.46 × 10^−3^
7	Apelin signaling pathway	18/137	1.05 × 10^−3^	1.25 × 10^−2^
8	Relaxin signaling pathway	17/129	1.39 × 10^−3^	1.53 × 10^−2^
9	AGE-RAGE signaling pathway in diabetic complications	14/100	2.00 × 10^−3^	2.01 × 10^−2^
10	Ras signaling pathway	25/232	2.37 × 10^−3^	2.22 × 10^−2^

## Data Availability

The datasets used and/or analyzed during the current study are available from the corresponding author upon reasonable request.

## Supplementary Materials

The following supporting information can be downloaded at: https://www.mdpi.com/article/10.3390/biomedicines11010069/s1, Table S1: Unique significant differential expression; Table S2: Clinical characteristics of MIBC from TCGA in each cluster; Figure S1: ROC curve analysis was performed for all 37 genes and all clusters.
