# Supplementary material for: Molecular Subtyping in Muscle-Invasive Bladder Cancer on Predicting Survival and Response of Treatment
Source: Biomedicines. 2022 Dec 27;11(1):69. doi: 10.3390/biomedicines11010069 (PMC9856180; doi:10.3390/biomedicines11010069)
Supplement: Supplementary file 1 [file biomedicines-11-00069-s001.zip › biomedicines-2107524-supplementary.pdf]

Supplementary data

Table S1. Unique significant differential expression.

Cluster	Genes
A	CPSF7,ALYREF,PABPC1L,ITGA2,EFNA1,KRT23,KRT16,F3,CCL5,SIK1B,G6PD,IRF1,S100A8,CD36,ACSL1
B	PPP2R2A,HK2,IGF1R,EP300,VHL,CAMK2G,EGLN3,SLC9A1,TSC2,NCOR1,TP53,ATF6B,KRT20,CREB3L2,PRKCD,HSPA1A,IRS1,CRKL,MAP3K5,RBL2,COL4A6,COL4A5,MET,IFNAR1,PRKAA1,GNG5,COL9A2,GNA15,MMP1,CAB39L,ADIPOR1,ELAVL1,ULK1,PATJ,TEAD3,FZD6,FRMD6,CAT,PGM2,PGLS,XIAP,NLRP1,ERBIN,EHMT2,SOD1,EIF4EBP2,TELO2,GNA11,GTF2I,EML4,VAV2,ELK4,MECOM,DUSP4,DUSP2,TAOK2,NF1,SGPL1,SPTLC2,ACER2,CCNG1,DBI,ACSL3,FABP4,SCP2,FABP5,ACOX1,UBC,PLIN2,HMGCS2,ACAA1,PLTP,SKIL,ZFHX3,PCGF3,TBX3,ID3,TCF3
C	ITGB5,TNC,FGF7,MYC,PDGFRB,VWF,ITGA1,COL6A3,ITGA7,ITGA5,CSF1R,LAMA4,THBS2,PPP2CB,FGFR1,CASC3,ICAM1,CCL2,STAT5B,CYBB,SELE,IL1B,BAX,PFKFB3,HMOX1,NOTCH2,PLN,PRKACA,RCAN1,ROCK1,ROCK2,SRF,MYLK,RGS2,MEF2D,PPP1R12A,IRAG1,GNAQ,MYL9,MMP9,HBEGF,STMN1,MAP2K3,DUSP5,DUSP3,IL1R1,CACNA1H,RAP1A,MAP3K20,CD14,FLNC,MAP4K4,GADD45B,GADD45A,NFKB2,ABL1,RAPGEF1,ITGB2,ENAH,PPP1R12B,ACTA2,SORBS1,RHOQ,CCN2,WWTR1,KLF2,APLNR,TNFAIP3,CXCL2,JUNB,DAB2IP,CFLAR,TNFRSF1B,BCL3,ANTXR2,ANTXR1,FOSL1,EGR3,LSP1,CXCL14,GRK2,CXCL12,UBE2I,PARP1,PLAU,RAB5B,RAB5C,ETS1,ETS2,RALBP1,PLA2G2A,RGL2,ARF6,DEGS1,LTBP1,DCN,GREM1,NBL1,SKP1,FBN1,CAB39,RBPJ,FHL1,STAT6,IL13RA1,IL6ST

Table S2. Clinical characteristics of MIBC from TCGA in each cluster.

	Cluster A	Cluster B	Cluster C	Total	p-value
	113	74	44	231	
Gender					
Male	83 (49.1)	57 (33.7)	29 (17.2)	169	
Female	30 (48.4)	17 (27.4)	15 (24.2)	62	0.4176
Age	69.57 ± 10.52	68.14 ± 10.25	69.53 ± 11.08		0.6536
Follow up time	2.40 ± 1.44	2.17 ± 2.07	2.24 ± 2.75		0.8528
Status					
Alive	83 (49.1)	57 (33.7)	29 (17.2)	169	
Dead	30 (49.2)	17 (27.9)	14 (23.0)	61	0.4176
AJCC pathological stage					
Stage II	35 (53.0)	21 (31.8)	10 (15.2)	66	

Stage III	46 (50.5)	31 (34.1)	14 (15.4)	91	
Stage IV	32 (43.2)	22 (29.7)	20 (27)	74	0.3255
Pathological tumor stage					
T2	38 (50.7)	22 (29.3)	15 (20.0)	75	
T3	57 (46.3)	43 (35.0)	23 (18.7)	123	
T4	15 (54.5)	9 (27.3)	6 (18.2)	33	0.8753
Pathological lymph node stage					
N0	75 (52.4)	46 (32.2)	22 (15.4)	143	
N1	12 (42.9)	9 (32.1)	7 (25.0)	28	
N2	18 (42.9)	11 (26.2)	13 (31.0)	42	
N3	2 (50.0)	2 (50.0)	0 (0.0)	4	
Nx	6 (42.9)	6 (42.9)	2 (14.3)	14	0.4392
Pathological metastasis stage					
M0	54 (46.6)	36 (31.0)	26 (22.4)	116	
M1	1 (20.0)	2 (40.0)	2 (40.0)	5	
Mx	58 (52.7)	36 (32.7)	16 (14.5)	110	0.3451
mRNA clustering					
Basal	49 (44.5)	26 (37.7)	11 (27.5)	86	
Luminal	56 (50.9)	42 (60.9)	29 (72.5)	127	
Neuronal	5 (4.5)	1 (1.4)	0 (0.0)	6	0.05412
Histological subtype					
Papillary	28 (56.0)	15 (30.0)	7 (14.0)	50	
Non-papillary	81 (48.8)	54 (32.5)	31 (18.7)	166	
Not determine	1 (33.3)	0 (0.0)	2 (66.7)	3	0.2016
Histological grade					
High grade	104 (49.5)	67 (31.9)	39 (18.6)	210	
Low grade	5 (62.5)	2 (25.0)	1 (12.5)	8	
Not determine	1 (100.0)	0 (0.0)	0 (0.0)	1	0.822
Lymphovascular invasion					
Yes	35 (43.2)	23 (28.4)	23 (28.4)	81	
No	46 (57.5)	27 (33.8)	7 (8.8)	80	
Not determine	29 (50.0)	19 (32.8)	10 (17.2)	58	0.03155

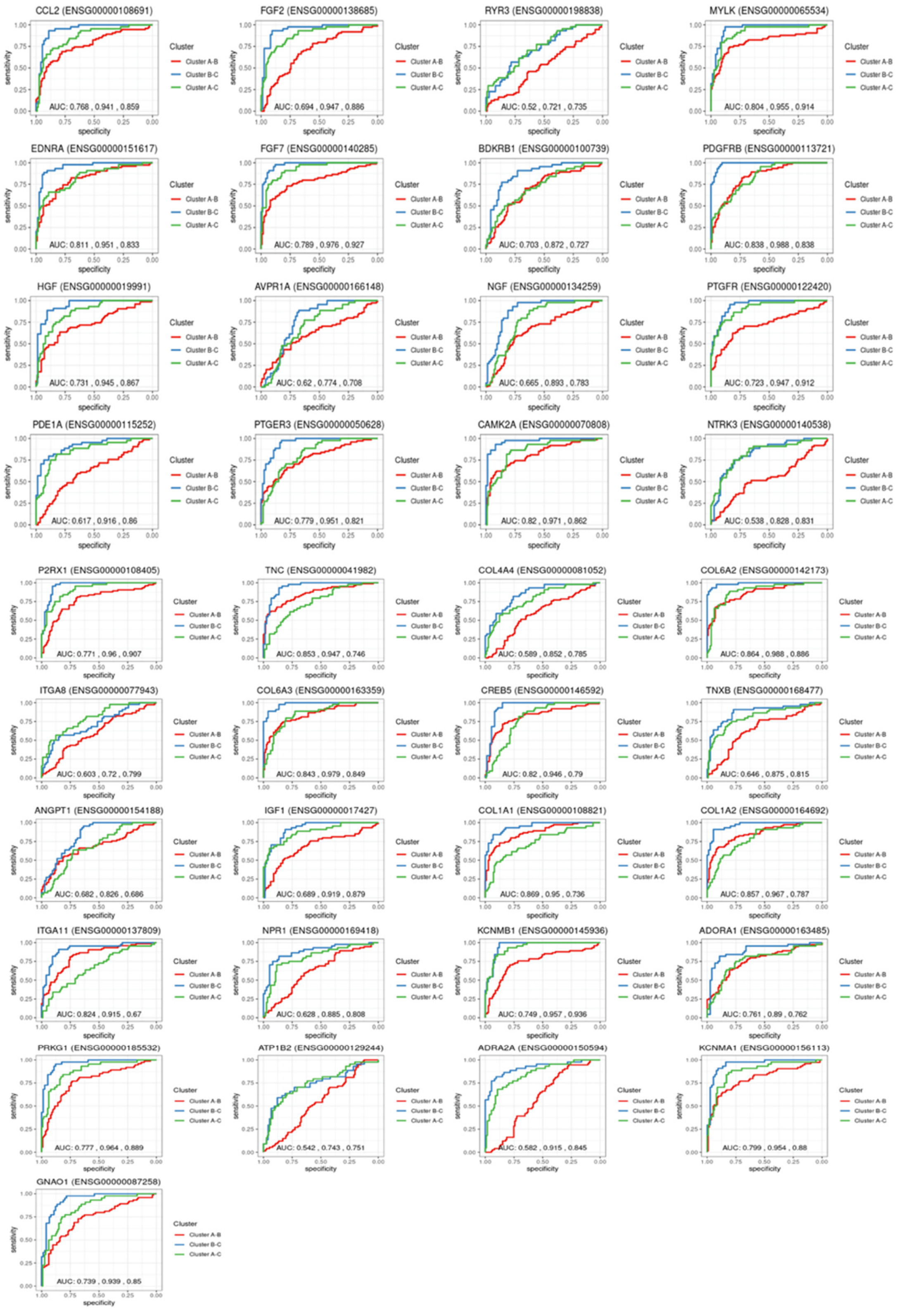

Figure S1 ROC curve analysis was performed for all 37 genes and all cluster. Interestingly, the corresponding areas under the ROC curve (AUCs) with the value more than 0.8, 0.9, and 0.95 were found in 33, 25, and 14 genes from 37 genes for cluster B.
